# An Up-to-Date Correlation of Epidemiological and Histopathological Characteristics of Basal Cell Carcinoma of the Skin in a County Hospital in Romania

**DOI:** 10.3390/dermatopathology9020023

**Published:** 2022-06-18

**Authors:** Iuliu Gabriel Cocuz, Maria Elena Cocuz, Adrian-Horațiu Sabău, Raluca Niculescu, Andreea Cătălina Tinca, Vlad Vunvulea, Corina Eugenia Budin, Mihaela Cornelia Șincu, Maria Cătălina Popelea, Ovidiu Simion Cotoi

**Affiliations:** 1 Doctoral School of Medicine and Pharmacy, “George Emil Palade” University of Medicine, Pharmacy, Sciences and Technology of Targu Mures, 540142 Targu Mures, Romania; iuliu.cocuz@umfst.ro (I.G.C.); mihaela.sincu02@gmail.com (M.C.Ș.); 2 Pathology Department, Mures Clinical County Hospital, 540011 Targu Mures, Romania; sabauhoratiu@gmail.com (A.-H.S.); niculescuralu@yahoo.com (R.N.); andreeatinca93@gmail.com (A.C.T.); popelea.maria@gmail.com (M.C.P.); ovidiu.cotoi@umfst.ro (O.S.C.); 3 Pathophysiology Department, “George Emil Palade” University of Medicine, Pharmacy, Sciences and Technology of Targu Mures, 540142 Targu Mures, Romania; cora_bud@yahoo.com; 4 Fundamental Prophylactic and Clinical Disciplines Department, Faculty of Medicine, Transilvania University of Brasov, 500003 Brașov, Romania; 5 Clinical Pneumology and Infectious Diseases Hospital of Brasov, 500118 Brasov, Romania; 6 Anatomy and Embryology Department, “George Emil Palade” University of Medicine, Pharmacy, Sciences and Technology of Targu Mures, 540142 Targu Mures, Romania; vlad.vunvulea@umfst.ro

**Keywords:** basal cell carcinoma, skin, nonmelanoma skin cancer, epidemiology, histopathology

## Abstract

*Background and Objectives*: Basal carcinoma of the skin (BCC) is part of the nonmelanoma skin cancer (NMSC) family and is the most frequently occurring type of skin cancer in humans. A combination of clinical and histopathological approaches is necessary in order to establish the best treatment regime for patients who have been diagnosed with this type of cancer. The objective of the present study was to establish the statistical value of prediction for certain sociodemographic characteristics (age category and environment of origin) and histopathological parameters of the subjects that could be related to the incidence of diagnosis with certain histopathological subtypes of BCC. *Materials and Methods*: In order to verify the veracity of the established research hypotheses, we conducted a retrospective study based on the histopathological reports of 216 patients who were treated at the Pathology Department of Mureș Clinical County Hospital. *Results*: Cystic BCC is higher in patients who are older than 71 years of age, and the superficial multicentric and keratotic subtypes are more frequently diagnosed in urban areas. Patients who have been diagnosed with the superficial multicentric BCC subtype are not usually very old in contrast to the patients who tend to be diagnosed with the cystic BCC subtype. The nodular BCC subtype is positively associated with ulceration (*p* = 0.004); the superficial multicentric BCC subtype is positively associated with intra- and peritumoral inflammatory infiltrate (*p* = 0.022, *p* = 0.034) and negatively associated with ulceration (r = −0.218, *p* = 0.001). The infiltrative BCC subtype is positively associated with ulceration (*p* = 0.021), and the keratotic BCC subtype is positively associated with peritumoral inflammatory infiltrate (*p* = 0.02). *Conclusions*: Depending on each patient’s epidemiological and sociodemographic data, a pattern can be established regarding the appropriate clinical and treatment approaches for that patient, which can be supported based on the implications of the histopathological diagnostic. This can lead to an improvement in the patient’s quality of life and increased satisfaction with the provided medical services.

## 1. Introduction

Basal cell carcinoma of the skin can be defined as a group of malignant tumors that are part of the nonmelanoma skin cancers (NMSC) that include basal cell carcinoma (BCC), squamous cell carcinoma (SCC), and metatypical (basosquamous) carcinoma [1,2,3]. The development of each specific carcinoma in this family depends on the proliferation of the basaloid cells or germinative cells, which may be organized into columns, cords, lobules, or bands. BCC appears more frequently in people over 40 years of age and in males [4]. BCC represents 75% of all of the NMSCs, and its incidence is increasing due to prolonged sun exposure and the increased age of the general population [5,6]. The incidence of BCC is not well established in Europe because the reporting system changes from one country to another, and even if a country has a cancer registry, the different BCC subtypes are not well reported [7,8].

Risk factors for BCC include different categories of various associated parameters such as UV radiation, which includes sun exposure due to specific professions as well as exposure that occurs due to leisure activities, but other risk factors also include age, gender, a family history of skin cancer, and individual skin phenotype, among others [4,9,10].

Histopathological diagnostics remains one of the most important tools for the management of BCC, and it continues to be important due to the fact that the quality of life of patients who have been diagnosed with skin cancer must be improved, even though the rate of mortality due to BCC is not very significant [11,12].

The main objective of our study is to analyze the characteristics of skin BCC, which can be subdivided into six additional target objectives:

1. The collection and analysis of data from histopathological bulletins from tissue samples obtained through surgical excision from subjects who have been diagnosed with BCC within the period of 2016–2020;
2. The establishment of the incidence of histopathological subtypes of BCC that have been diagnosed and their comparison according to the years of diagnosis;
3. The analysis of the degree of occurrence of histological and histopathological parameters (ulceration, excision within surgical safety limits, intra- and peritumoral inflammatory infiltrate, perineural invasion, and perivascular invasion) in each subtype of diagnosed BCC;
4. The establishment of the statistical value of prediction of certain sociodemographic characteristics of the subjects (age category and environment of origin) on the incidence of diagnosis with certain histopathological subtypes of BCC;
5. The establishment of a correlation between the age of the subjects and the incidence of diagnosis with certain histopathological subtypes of BCC;
6. The establishment of correlations between certain histological and histopathological parameters of the analyzed tumors and the different histopathological subtypes of diagnosed BCC.

Based on these six target objectives, we aim to determine the veracity of three general research hypotheses, the first of which comprises the two following hypotheses:

**Hypothesis 1 (H1).**  *There are significant differences in the incidence of certain histopathological subtypes of BCC, and these differences depend on certain sociodemographic characteristics.*

Specific hypotheses:

**Hypothesis 1.1 (H1.1).**  *The incidence of certain histopathological subtypes of BCC differs significantly depending on the age category of the subjects.*

**Hypothesis 1.2 (H1.2).**  *The incidence of certain histopathological subtypes of BCC differs significantly depending on the subject’s environment of origin.*

**Hypothesis 2 (H2).**  *There are statistically significant relationships between the age of the subjects and the incidence of certain histopathological subtypes of BCC that are diagnosed.*

**Hypothesis 3 (H3).**  *There are statistically significant relationships between certain histological and histopathological parameters of the analyzed tumor formations and different histopathological subtypes of BCC that are diagnosed.*

## 2. Materials and Methods

In order to determine the veracity of the established research hypotheses, a retrospective study was performed between 2016 and 2020 that involved the analysis of data from histopathological bulletins of skin tissue samples that were obtained through surgical excision and that were received from the departments of Dermatology, Plastic Surgery, or General Surgery in the Pathology Department of Mureș County Clinical Hospital. Tissue samples came from outpatient visits, day hospitalizations, or continuous hospitalizations and were obtained from a total of 216 patients.

The inclusion criteria for the patients to be enrolled in the study was the diagnosis of skin BCC established by our pathology department and the period when the diagnosis was established. The exclusion criteria were other histopathological diagnoses and a BCC diagnosis that was established outside of the study period. All of the histopathological diagnostics that met the inclusion criteria were included in the study. The limitations of the study were represented by the fact that the classification of BCC presented in this study is based on the data from the histopathological reports in our Pathology Department on a retrospective basis.

Sample analysis was conducted by embedding the tissues in paraffin, and the tissues were stained with hematoxylin–eosin; through this procedure, histopathological diagnoses of basal cell carcinoma (BCC) on the skin were established. All tissue samples came from complete excision of the lesion. The following factors and parameters were analyzed:

- Year of diagnosis;
- Age of the patient at the time of diagnosis;
- Area where the patient lived at the time of diagnosis;
- Gender of the patient;
- Tumor ulceration and the presence or the absence of a hemorrhage or inflammation area on the surface of the tumor;
- Excision within surgical safety limits and whether or not the tumor resection margins were invaded by the tumor in order to determine whether it had been completely excised and whether any tumoral tissue remained inside of the patient;
- Intratumorally inflammatory infiltrate and whether or not any inflammatory infiltrate was present inside of the tumor;
- Peritumoral inflammatory infiltrate and where there was any inflammatory infiltrate around the tumor;
- Perineural/perivascular invasion of tumor cells.

After the data were collected, they were coded with their nominal and ordinal values so that they could be entered into SPSS (Statistical Package for the Social Sciences). The collected data were coded in accordance with each type of data analyzed.

The statistical analysis consisted of the use of frequency and comparative tables and graphs for each of the six histopathological subtypes of basal cell carcinoma that were diagnosed and the five histological and histopathological parameters of the analyzed tumors; *t*-tests were conducted for independent samples, and Pearson bivariate correlations were conducted to determine the veracity of the research hypotheses.

To verify the first research hypothesis and to measure significant differences in the incidence of certain histopathological subtypes of basal cell carcinoma, *t*-tests were used for independent samples to analyze the differences between the following subgroups:

- Subjects under the age of 70 and those over the age of 71;
- Subjects from urban and rural areas.

To verify the second research hypothesis, Pearson bivariate correlations were used to analyze the existence of statistically significant relationships between the age of the subjects and the incidence of certain histopathological subtypes of BCC that had been diagnosed.

To verify the latest research hypothesis, Pearson bivariate correlations were also used to analyze the existence of statistically significant relationships between each of the five histological and histopathological parameters of the analyzed tumors (ulceration, excision within surgical safety limits, intra- and peritumoral inflammatory infiltrate, perineural invasion, and perivascular invasion) and the six histopathological subtypes of BCC that had been diagnosed.

## 3. Results

### 3.1. Sociodemographic and Epidemiological Data

To determine the veracity of the research hypotheses, the data from the histopathological bulletins of the tissue samples that had been obtained by surgical excision from 216 subjects who had been diagnosed with basal cell carcinoma between 2016 and 2020 were analyzed.

Table 1 shows the distribution of these subjects according to the year of diagnosis, gender, age group, and environment of origin.

### 3.2. Descriptive Results

Table 2 shows the number of histopathological subtypes that were diagnosed in the 216 subjects as well as the BCC category, which was determined according to this number.

Table 3 shows the frequency of all of the histopathological basal cell carcinoma subtypes that were diagnosed in the 216 subjects, as well as a comparison of their incidence based on the year of diagnosis.

Table 4 shows the presence of the five histological and histopathological parameters of the tumor formations that were analyzed in each of the six basal cell carcinoma subtypes that were diagnosed in the 216 subjects as well as a comparison of their incidence based on the year of diagnosis.

### 3.3. Research Hypothesis Interpretation

#### 3.3.1. General Research Hypothesis 1

There are significant differences in the incidence of certain histopathological subtypes of BCC that are dependent on certain sociodemographic characteristics of the subjects.

This hypothesis refers to the diagnostic incidence of subjects with the six histopathological basal cell carcinoma subtypes (nodular, superficial multicentric, adenoid, cystic, infiltrative, and keratotic), depending on their sociodemographic characteristics.

We chose the age group to which each subject belonged and their environment of origin as two sociodemographic characteristics representing differentiating criteria, and thus, we divided this general hypothesis into two specific hypotheses. The two specific hypotheses refer to sociodemographic characteristics, according to which we assumed that there would be significant differences in terms of the diagnostic incidence of subjects with the six histopathological BCC subjects.

##### Specific Research Hypothesis 1.1 (H1.1)

The incidence of certain histopathological subtypes of basal cell carcinoma differs significantly depending on the age of the subject.

**Figure 1:**
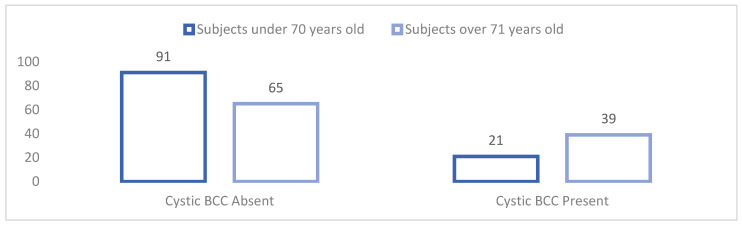
Significant differences between subjects under 70 years of age and over 71 years of age in the incidence of diagnosis with the cystic BCC subtype.

In order to verify this hypothesis, a *t*-test was used for independent samples in order to measure the existence of significant differences in the incidence of diagnosis with the six histopathological basal cell carcinoma subtypes that were diagnosed between subjects under 70 years of age (*n* = 112) and those over 71 years of age (*n* = 104). The values, significance thresholds of t, and the average incidence of the six histopathological basal cell carcinoma subtypes with which subjects under 70 years of age and those over 71 years of age were diagnosed are shown in Table 5 and Figure 1.

##### Specific Research Hypothesis 1.2 (H1.2)

The incidence of certain histopathological BCC subtypes differs significantly depending on the environment of origin of the subjects.

**Figure 2:**
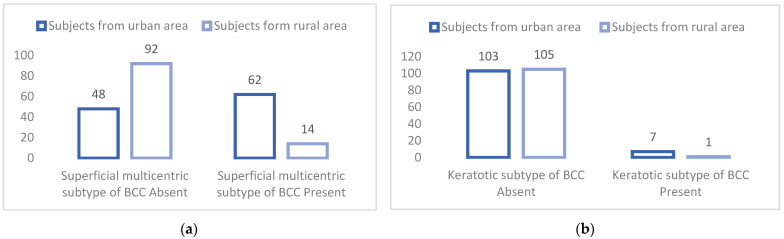
(**a**) Significant differences between urban and rural subjects in terms of the incidence of diagnosis with superficial multicentric BCC subtype. (**b**) Significant differences between urban and rural subjects in the incidence of diagnosis with keratotic BCC subtype.

In order to verify this hypothesis, a *t*-test was used for independent samples in order to measure the existence of significant differences in the incidence of diagnosis with the six histopathological basal cell carcinoma subtypes between urban subjects (*n* = 110) and those living in a rural environment (*n* = 106). The values, significance thresholds of *t*, and the average incidence of the six histopathological BCC subtypes with which urban and rural subjects were diagnosed are shown in Table 6 and in Figure 2a,b.

#### 3.3.2. General Research Hypothesis 2

There are statistically significant relationships between the age of the subjects and the incidence of certain histopathological subtypes of BCC that were diagnosed.

**Figure 3:**
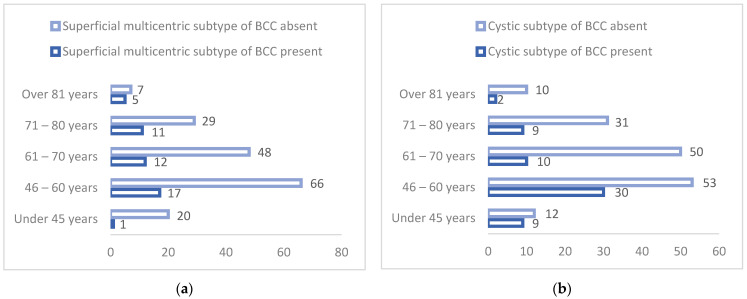
(**a**) Incidence of superficial multicentric BCC subtype diagnosis correlated with the age of the subject. (**b**) Incidence of cystic BCC subtype diagnosis correlated with the age of the subjects.

In order to verify the veracity of this hypothesis, we used Pearson bivariate correlations to determine the existence of statistically significant relationships between the age of the subjects and the incidence of their diagnosis with the six histopathological BCC subtypes: nodular, superficial multicentric, adenoid, cystic, infiltrative, and keratotic. The values and significance thresholds of the correlation coefficients are shown in Table 7 and Figure 3a,b.

Correlations were calculated based on the values related to the coding of each histopathological BCC subtype, with 1 = absent and 2 = present.

#### 3.3.3. General Research Hypothesis 3

There are statistically significant relationships between certain histological and histopathological parameters of the analyzed tumor formations and the different histopathological subtypes of BCC that were diagnosed.

In order to determine the veracity of this hypothesis, we used Pearson bivariate correlations to determine the existence of statistically significant relationships between each of the five histological and histopathological parameters of the analyzed tumors, ulceration, excision within surgical safety limits, intra-inflammatory infiltrate and peritumoral, perineural invasion, and perivascular invasion, and the incidence of diagnosis with each of the histopathological BCC subtypes (nodular, superficial multicentric, adenoid, cystic, infiltrative, and keratotic).

The correlations between each parameter of the analyzed tumor formations and the incidence of diagnosis with the six histopathological basal cell carcinoma subtypes mentioned above are presented in Table 8.

## 4. Discussion

Nonmelanoma skin cancers (NMSC) are divided into the categories of basal cell carcinoma (BCC), squamous cell carcinoma (SCC), and metatypical (basosquamous) carcinoma. From these categories, BCC is the most frequent type of NMSC that is found worldwide, making it the skin cancer type that is the most frequently observed [13]. The incidence of BCC cannot be estimated correctly due to the fact that many countries either do not have cancer registries or because the NMSC are not always recorded individually according to their names [14].

The first part of our study presented some demographic characteristics that may be attributed to the incidence of BCC among the Romanian population, and especially in those members of the population who visit the Pathology Department of the Mureș Clinical County Hospital, which is where the analyzed samples were collected (though some were also collected from neighboring areas).

Table 1 shows that more than one-third of the subjects (36.5%) were diagnosed with basal cell carcinoma (BCC) in 2019, and nearly one-quarter (23%) were diagnosed with BCC in 2018. This represents an increase of 216% in the number of BCC cases that were diagnosed in 2019 compared to in 2016; this was followed by a decrease in the number of BCC cases that were diagnosed in 2020 of 59.5% compared to the previous year. In 2021, Cocuz et al. reported that there was a difference in the number of cases of BCC diagnosed in 2019 compared to 2020 because of the COVID-19 pandemic, which led to fewer surgical excisions of this type of NMSC [15].

The COVID-19 pandemic strongly influenced the way that patients were treated in terms of surgery, especially oncological patients. The expectation is that the number of skin cancers that will be diagnosed will increase. Specifically, there will not only be an increase in the number of skin BCC diagnosed, but there will also be an increase observed in the other NMSC categories as well as in the number of melanoma cases. Unfortunately, we are prepared to see cases of skin cancer that are in more advanced stages due to the fact that patients did not receive adequate treatment on time, as well as because skin cancer management during the diagnosis and treatment phases was affected by the transformation of hospitals in to hospitals that were meant for the exclusive treatment of COVID-19. The Omicron variant of the SARS-CoV-2 virus may further the diagnosis of NMSCs by overloading healthcare systems worldwide [15,16].

The gender distribution of the entire group of subjects is approximately equivalent, with 54% of the cases being male patients and 46% being female patients. In terms of the gender distribution of the subjects, depending on the year when the patient was diagnosed with BCC, most of the male subjects were diagnosed in 2019 (39%), and most of the female participants were also diagnosed in 2019 (34%). In their research, Fidelis et al. mentioned that that there was a higher incidence of BCC in men than there was in women in Brazil in 2021 [17]; this is in direct contrast with the higher incidence in men determined by Lang et al. [18]. Miolo et al. mentioned that that 218 BCC cases were diagnosed in people who were 66 years of age and older in their study [19].

Regarding the age distribution of the subjects depending on the year in which they were diagnosed with BCC, most of the participants who were under 70 years old were diagnosed in 2019 (37%), and most of the patients over the age of 71 were diagnosed in 2019 (37%), 2018 (24%), and 2020 (17%). Based on Lang et al., most cases of BCC are diagnosed in individuals who are 71 years of age and over. This is also in accordance with the results that were obtained in our study [13].

The distribution of BCC cases based on the origin of the patients at the level of the study group was approximately equivalent, with 51% of the individuals coming from urban areas, and 49% of the included individuals coming from rural areas. Regarding the environmental distribution of the subjects based on the year in which they were diagnosed with BCC, most of the people who were living in urban areas were diagnosed in 2019 (38%), and most of the people who were living in rural areas were also diagnosed in 2019 (35%). In their study, Carcin et al. observed a higher incidence of BCC in urban areas such as Dublin, Cork, Galway, and Waterford in Ireland, which can also be seen in our study area of Târgu Mureș [13]. Targu Mureș has a very well-developed chemical industry. As Torchia et al. and Bowra et al. mentioned in their research, long-term exposure to chemicals may lead to skin cancers, especially BCC. Due to this chronic exposure, it can be said that some types of BCC may be more frequent in urban areas [20,21].

There are various BCC subtypes, with many lesions only presenting one BCC subtype; however, there have been cases where more than one BCC subtype has been found in an individual lesion. Our 216 subjects were divided into two main categories: those demonstrating only one BCC subtype in their histopathological diagnostic, and those with a mixed BCC type. A total of 60% of the 216 subjects were diagnosed with mixed BCC, presenting several histopathological subtypes of this neoplastic disease, while 40% were diagnosed with simple BCC, presenting a single histopathological subtype. One third of the subjects (35%) presented with two subtypes, and 20% presented with three histopathological BCC subtypes. Of the subjects with a mixed diagnosis, almost half (47%) represented the nodular BCC subtype, which was the most frequent one, a fact that was also mentioned by Chung in his research, but this type occurred less frequently in our study [22]. Approximately 15% demonstrated the cystic BCC subtype, 17% presented with the superficial multicentric BCC subtype, and 11% were diagnosed with the adenoid BCC subtype. Regarding the comparison of the annual incidence of all of the BCC subtypes, it was observed that over a third were diagnosed in 2019, 26% were diagnosed in 2018, and 18% were diagnosed in 2020. BCC had the highest incidence in 2019, followed by a strong decrease in 2019. Comparing the annual incidence at the level of each histopathological subtype of BCC among the six types, we observed that superficial multicentric BCC had the highest incidence in 2018, while nodular, adenoid, cystic, infiltrative, and keratotic BCC had the highest incidence of diagnosis in 2019.

The second part of our study was targeted on proving our three established research hypotheses. Taking the hypothesis that there are significant differences between subjects under 70 years of age and those over 71 years of age into consideration, we found that there were significant differences in terms of the incidence of the cystic BCC subtype. Figure 1 shows this histopathological BCC subject graphically, in which there are significant differences in terms of the incidence of diagnosis that are dependent on the age of the subject. The incidence of diagnosis with the cystic BCC subtype is significantly higher in subjects over 71 years of age compared to subjects who are under 70 years of age. On average, subjects who are over the age of 71 are diagnosed with cystic BCC more frequently than those who are under the age of 70. Considering that the incidence of diagnosis with one of the six histopathological BCC subtypes and that the cystic subtype differs significantly depending on the age of the subjects, we can confirm specific research Hypothesis 1.1. Analyzing the data from Table 6, we are able to determine that there are significant differences between the environment from which each subject is from in terms of the incidence of diagnosis with the superficial multicentric BCC subtype and the keratotic BCC subtype. Figure 2a,b graphically represent the two histopathological subtypes of BCC in which there are significant differences in the incidence of diagnosis that are dependent on where the subjects are from. Considering the diagnostic incidence with two of the six histopathological BCC subtypes, and that the incidence of the diagnosis of the superficial multicentric and keratotic subtypes differs significantly depending where the subjects are from, we are able to confirm Hypothesis 1.2. The confirmation of Hypotheses 1.1. and 1.2. lead to the confirmation of the first general research hypothesis, attesting that there are significant differences in the incidence of certain histopathological subtypes of basal cell carcinoma that are dependent on the age of the patient and where they are from.

Taking Table 7 into consideration, we are able to observe that the age of the subjects correlates significantly with the diagnostic incidence of two of the six histopathological basal cell carcinoma subjects: the superficial multicentric BCC subtype, and the cystic BCC subtype. The first correlation is negative, whereas the second correlation is positive. Figure 3a,b show that the age of the subjects demonstrated a significant negative correlation with the incidence of the superficial multicentric BCC subtype. This means that the older the subjects are, the lower the probability of being diagnosed with superficial multicentric BCC is. We also discovered that the age of the subjects demonstrates a significant positive correlation with the incidence of the cystic BCC subtype, which means that the older the subjects are, the more likely they are to be diagnosed with cystic BCC. Given that the age of the subjects correlates significantly with the incidence of diagnosis for two of the six histopathological basal cell carcinoma subtypes and correlates specifically with the superficial multicentric BCC subtype and cystic subtype BCC, we can confirm the second general research hypothesis.

Ulceration occurs in more than half of the cases of infiltrative subtype BCC alone, which is in direct accordance with Tanase, who states that the infiltrative subtype of BCC is the most common subtype that is able to present with ulcerations [23].

Ulceration showed a highly significant and positive correlation with the incidence of the diagnosis of subjects with the nodular BCC subtype and with the infiltrative BCC subtype. The higher the incidence at which subjects were diagnosed with the nodular and infiltrative BCC subtypes, the higher the probability of ulceration in this histopathological subtype of BCC. It can be said that most diagnoses of the nodular and infiltrative subtypes of BCC are associated with ulceration. Ulceration showed a significant and negative correlation with the incidence of the diagnosis of subjects with superficial multicentric BCC, which means that the higher the incidence at which patients are diagnosed with superficial multicentric BCC, the lower the probability of ulceration. Most diagnoses of the superficial multicentric subtype BCC are not associated with ulceration, as proven by our results.

Regarding excision within surgical safety limits, it can be seen that surgical excision safety was possible in more than half of the cases in all of the six subtypes of BCC that were diagnosed. In their systemic review from 2020, Quazi et al. observed that excision within surgical safety limits depends on the subtype of BCC [24]. During our study, as shown in Table 8, we observed that excision within surgical safety limits correlates significantly with diagnostic incidence of one of the six histopathological basal cell carcinoma subtypes, and is specifically correlated with BCC of the cystic subtype. The higher incidence at which subjects are diagnosed with cystic BCC, the higher the likelihood that tumor excision will be performed within surgical safety limits within this histopathological subtype of BCC. From this, we can say that most diagnoses of cystic subtype BCC are associated with excisions being within surgical safety limits.

As shown in our results, intratumoral inflammatory infiltrate occurred in more than half of the cases of the superficial multicentric BCC subtype and the keratotic BCC subtype, whereas peritumoral inflammatory infiltrate occurred in more than half of the cases for the superficial multicentric BCC subtype. Regarding the intratumorally inflammatory infiltrate, it is seen that it correlates significantly with the diagnostic incidence of two of the six histopathological basal cell carcinoma subtypes, specifically with superficial multicentric BCC subtype. Most diagnoses of the superficial multicentric subtype BCC are associated with intratumorally inflammatory infiltrate. Peritumoral inflammatory infiltrate correlates significantly with the diagnostic incidence of three of the six histopathological basal cell carcinoma subtypes, especially with the superficial multicentric and keratotic BCC subtypes. This means that most diagnoses of superficial multicentric and keratotic BCC subtypes are associated with peritumoral inflammatory infiltrate.

Neither perineural nor perivascular invasion have an incidence in more than half of the cases of any of the diagnosed BCC subtypes.

The data that were analyzed here showed the existence of statistically significant relationships between five of the histological and histopathological parameters of the analyzed tumors, specifically ulceration, excision within surgical safety limits, and intra- and peritumoral inflammatory infiltrate, and the different histopathological subtypes of basal cell carcinoma that were diagnosed, leading to the confirmation of the third general research hypothesis.

## 5. Conclusions

The present study confirmed the research hypotheses that were established and emphasized the importance of taking the specific particularities of each individual patient starting from their age into consideration before arriving at an adequate diagnosis. The incidence of the cystic subtype of BCC is statistically higher in patients who are older than 71 years of age. In terms of environment, the superficial multicentric and keratotic BCC subtypes are found more often in patients from urban areas. The patients who had been diagnosed with the superficial multicentric BCC subtype tended not to be very old, which was in contrast with the patients who were diagnosed with the cystic BCC subtype, who tended to be older. The relationships between the statistically significant association between the diagnosed basal cell carcinoma subtypes and the histological and histopathological parameters that are present have shown that the nodular BCC subtype is positively associated with ulceration, the superficial multicentric BCC subtype is positively associated with intra- and peritumoral inflammatory infiltrate; the cystic BCC subtype is positively associated with excision within surgical safety limits; the infiltrative BCC subtype is positively associated with ulceration; and the keratotic BCC subtype is positively associated with peritumoral inflammatory infiltrate. In conclusion, using the epidemiological and sociodemographic data of a patient can create a pattern for clinical and treatment approaches. This can lead to improvements in patient quality of life and increased satisfaction regarding the medical services provided.

## Figures and Tables

**Table 1:** Distribution of subjects according to the year of diagnosis, gender, age group, and the environment of origin.

Characteristic/Year		2016	2017	2018	2019	2020	Total
Male	Frequency (*n*)	15	13	24	45	19	116
	Percentage (%)	13%	11%	21%	39%	16%	100%
Female	Frequency (*n*)	14	18	25	41	14	112
	Percentage (%)	12.5%	16%	22%	37%	12.5%	100%
Under 70 Years	Frequency (*n*)	14	18	25	41	14	112
	Percentage (%)	12.5%	16%	22%	37%	12.5%	100%
Over 70 Years	Frequency (*n*)	11	12	25	38	11	104
	Percentage (%)	10.5%	11.5%	24%	37%	17%	100%
Urban Area	Frequency (*n*)	13	12	31	42	12	110
	Percentage (%)	12%	11%	28%	38%	11%	100%
Rural Area	Frequency (*n*)	12	18	19	37	20	106
	Percentage (%)	11%	17%	18%	35%	19%	100%

**Table 2:** Distribution of the number of histopathological subtypes of BCC diagnostics.

No. of Subtypes of BCC	No. of Subtypes of BCC		BCC Category		No. of Subtypes of BCC
	Frequency (*n*)	Percentage (%)	Frequency (*n*)	Percentage (%)	
1 subtype of BCC	86	40%	86	40%	Simple BCC
2 subtypes of BCC	76	35%	130	60%	Mixed BCC
3 subtypes of BCC	43	20%			
4 subtypes of BCC	11	5%			
Total	216	100%	216	100%	

**Table 3:** Distribution of all histopathological subtypes of BCC diagnosed and comparison of their annual incidence.

Histological Subtypes of BCC Diagnosed	2016	2017	2018	2019	2020	Total	
						Frequency (*n*)	Percentage (%)
Nodular BCC	22	27	46	70	28	193	47%
Adenoid BCC	1	7	9	16	11	44	11%
Superficial Multicentric BCC	5	4	28	26	13	76	18%
Cystic BCC	2	5	16	24	13	60	15%
Infiltrative BCC	2	2	7	11	8	30	7%
Keratotic BCC	2	1	2	3	0	8	2%
Total	34	46	108	150	73	411	100%

**Table 4:** Presence of histological and histopathological parameters of the tumor formations analyzed in each of the six subtypes of basal cell carcinoma that were diagnosed.

Histopathological Subtypes of BCC Diagnosed	Frequency of BCC Subtype Diagnostic	Ulceration (*n*)	Excision in Surgical Limits (*n*)	Intratumoral Inflammatory Infiltrate (*n*)	Peritumoral Inflammatory Infiltrate (*n*)	Perineural Invasion/Perivascular Invasion (*n*)	
Nodular BCC	193	85	140	65	68	1	1
Adenoid BCC	44	20	32	11	13	0	0
Superficial Multicentric BCC	76	17	40	42	42	2	2
Cystic BCC	60	24	50	23	24	1	1
Infiltrative BCC	30	18	21	9	11	0	0
Keratotic BCC	8	3	5	6	3	0	0
Total	411	167	298	156	161	4	4

**Table 5:** Comparison of the incidence of the six histopathological BCC subtypes according to the age of the subjects.

BCC Subtypes	*t*	Sig. Two-Tailed	Average of the Incidence of BCC Subtypes *	
			under 70 Years (*n* = 112)	over 71 Years (*n* = 104)
Incidence of nodular BCC subtype	1.83	0.069	1.86	1.93
Incidence of superficial multicentric BCC subtype	1.28	0.252	1.19	1.13
Incidence of adenoid BCC subtype	0.27	0.784	1.20	1.21
Incidence of cystic BCC subtype	3.10	0.002	1.19	1.38
Incidence of infiltrative BCC subtype	0.22	0.828	1.13	1.14
Incidence of keratotic BCC subtype	1.52	0.130	1.02	1.06

*t* = value of *t*; sig. (two-tailed) = 5% significance level of *t* (confidence interval is 95%). * Incidence averages were obtained by coding each histopathological BCC subtype, with 1 = absent and 2 = present.

**Table 6:** Comparison of the incidence of the six histopathological BCC subtypes depending on whether the subjects were from an urban or rural environment.

BCC Subtypes	*t*	Sig. Two-Tailed	Average of the Incidence of BCC Subtypes *	
			Urban (*N* = 110)	Rural (*N* = 106)
Incidence of nodular BCC subtype	1.01	0.314	1.87	1.92
Incidence of adenoid BCC subtype	0.14	0.891	1.20	1.21
Incidence of superficial multicentric BCC subtype	2.01	0.048	1.22	1.12
Incidence of cystic BCC subtype	1.01	0.283	1.25	1.31
Incidence of infiltrative BCC subtype	0.28	0.777	1.15	1.13
Incidence of keratotic BCC subtype	2.15	0.033	1.06	1.01

*t* = value of *t*; sig. (two-tailed) = 5% significance level of *t* (confidence interval is 95%). * Incidence averages were obtained by coding each histopathological BCC subtype, with 1 = absent and 2 = present.

**Table 7:** Correlation of the age of the subjects with the diagnostic incidence with the six histopathological BCC subtypes.

	Correlation Coefficient (*r*)	Significance Threshold (*p*) Two-Tailed
Incidence of nodular BCC subtype	0.079	0.249
Incidence of adenoid BCC subtype	0.032	0.640
Incidence of superficial multicentric BCC subtype	−0.134	0.110
Incidence of cystic BCC subtype	0.175 *	0.010
Incidence of infiltrative BCC subtype	−0.028	0.686
Incidence of keratotic BCC subtype	0.097	0.157

*r* = value of the correlation coefficient; *p* = significance level of *r*; * = *r* is significant when *p* < 0.05 (the probability of obtaining this correlation is <0.05).

**Table 8:** Correlation of the histopathological parameters with the incidence of the six histopathological BCC subtypes.

Parameter/Subtype of BCC	Nodular BCC		Adenoid BCC		Superficial Multicentric BCC		Cystic BCC		Infiltrative BCC		Keratotic BCC	
	*r*	*p*	*r*	*p*	*r*	*p*	*r*	*p*	*r*	*p*	*r*	*p*
Ulceration	0.195	0.004	0.049	0.478	−0.218	0.001	0.009	0.891	0.157	0.021	−0.013	0.850
Excision in surgical limits	0.050	0.463	0.011	0.874	−0.110	0.322	0.159	0.019	−0.16	0.819	−0.040	0.555
Intratumoral inflammatory infiltrate	−0.603	0.353	−0.103	0.130	0.124	0.022	0.047	0.492	−0.040	0.560	0.011	0.867
Peritumoral inflammatory infiltrate	−0.053	0.439	−0.069	0.312	0.124	0.034	0.050	0.463	0.005	0.946	0.159	0.020
Perineural invasion/perivascular invasion	−0.123	0.070	−0.049	0.475	0.101	0.139	0.048	0.483	−0.039	0.570	−0.019	0.782

*r* = value of the correlation coefficient; *p* = significance level of *r*; correlations were calculated based on the values related to the coding of each histopathological BCC subtype, with 1 = absent and 2 = present.

## Data Availability

All data produced here are available upon request.
